# Juvenile Idiopathic Arthritis With Epstein-Barr Virus-Associated Smooth Muscle Tumor in a 6-Year-Old Girl: A Rare Case Report

**DOI:** 10.3389/fped.2021.680113

**Published:** 2021-06-18

**Authors:** Zhijuan Kang, Juan Xu, Zhihui Li

**Affiliations:** ^1^Department of Nephrology and Rheumatology of Hunan Children's Hospital, Changsha, China; ^2^Academy of Pediatrics of University of South China, Changsha, China

**Keywords:** epstein barr virus, smooth muscle tumor, juvenile idiopathic arthritis, children, hip joint

## Abstract

Herein, we reported a rare case of Epstein-Barr virus-associated smooth muscle tumor (EBV-SMT) combined with juvenile idiopathic arthritis (JIA) in a 6-year old girl without HIV, organ transplantation, or congenital immunodeficiency. The patient suffered from pain in the bilateral hip joints, which drastically affected her physical activity. Consequently, she was diagnosed with JIA (September 2019). She was given methotrexate and methylprednisolone pills *via* oral route and a subcutaneous injection of Recombinant Human Tumor Necrosis Factor-α Receptor II;lgG Fc Fusion Protein for 4 weeks that successfully relieved the pain. In May 2020, the pain reoccurred and was accompanied by occasional headaches. After extensive pathological examination, the patient was diagnosed with EBV-SMT. The imaging examinations after admission showed multiple lesions in the skull, lungs, and vertebral body. Biopsy of the L2 vertebral body was then performed to clarify the diagnosis. Finally, the *in-situ* hybridization of the tumor of the lumbar vertebrae suggested a non-HIV/transplantation-related EBV-SMT. Consequently, the patient received surgery without chemotherapy and radiotherapy, after which her conditions improved.

## Background

Epstein-Barr virus-associated smooth muscle tumor (EBV-SMT) is an immunosuppression-related rare tumor that usually occurs in patients with acquired immune deficiency syndrome (AIDS), organ transplantation, or congenital immunodeficiency. Cases with EBV-SMT combined with juvenile idiopathic arthritis (JIA) have not been reported thus far. Herein, we report a single case of EBV-SMT combined with JIA in a 6-year-old patient without HIV, organ transplantation, or congenital immunodeficiency.

## Case Presentation

A 6-month-old girl presented to our clinic with bilateral hip joint pain, which began 1 year prior to her admission. The pain drastically affected her physical activity and was more evident in the morning. No other symptoms were presented. She then received Plain MRI and enhanced scanning of the hip joints, which revealed a small amount of effusion within the bilateral joints. Both colored ultrasound examination of retroperitoneum and Plain CT scanning of the chest and abdomen showed no evident abnormalities; thus, no special treatment was given to the child at that time.

The child still complained of recurrent hip pain, and her parents treated her with physical therapy, with no significant improvement. In September 2019, the girl was admitted to our clinic for the first time, and the Plain MRI and enhanced scanning showed a small amount of bilateral hip joint effusion. Cytological examinations of the bone marrow were normal. However, her parents treated the child with irregular traditional Chinese medicine and still did not see any significant improvement.

**Figure 1:**
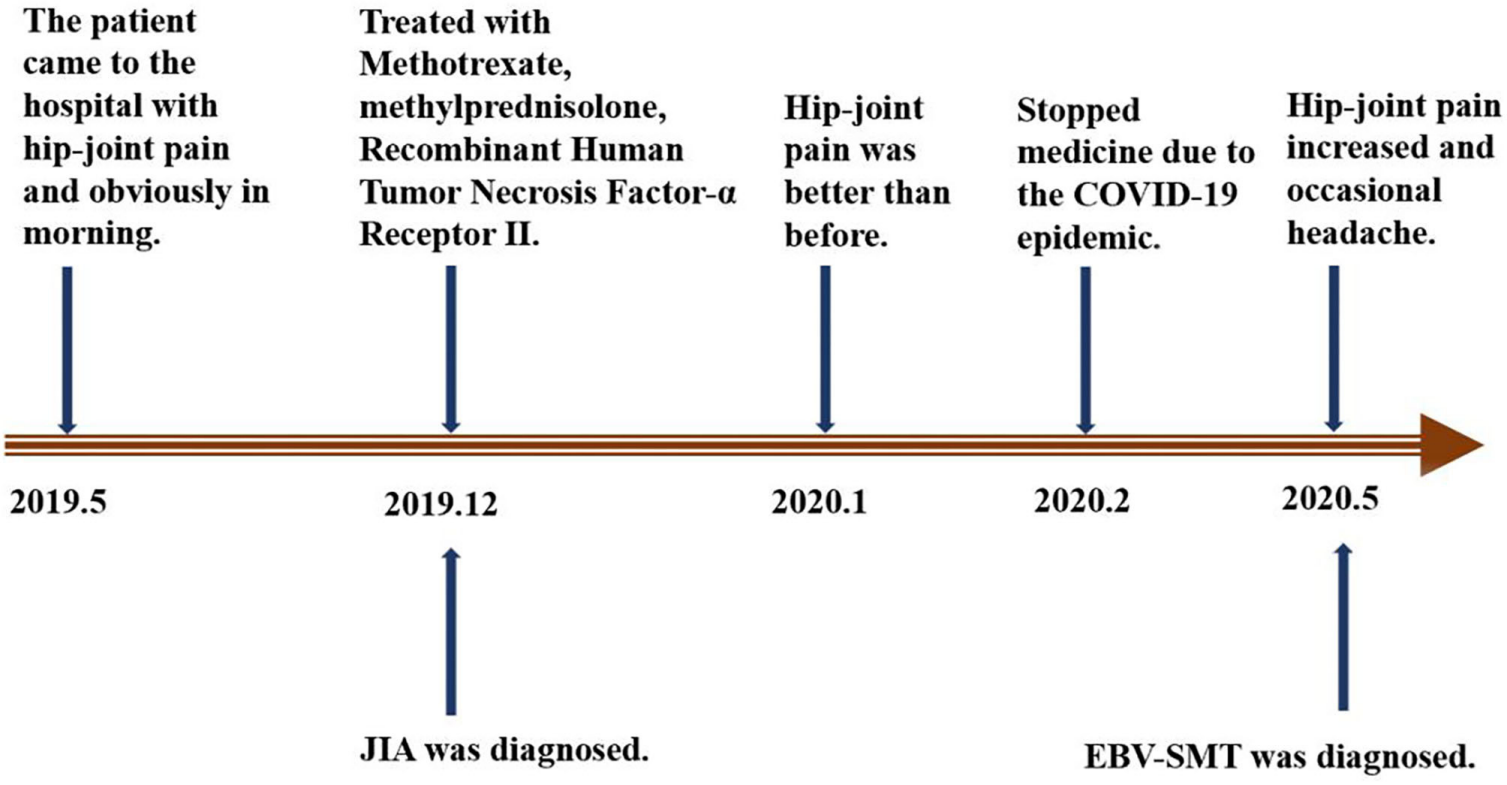
Diagram of the disease progression.

In December 2019, the girl was admitted to another hospital and diagnosed with JIA. At that time, EBV-DNA quantification was normal. From then on, she received oral administration of methotrexate (MTX, 14 mg/m^2^, once per week), methylprednisolone pills (0.75 mg/kg/d), and subcutaneous injections of Recombinant Human Tumor Necrosis Factor-α Receptor II; lgG Fc Fusion Protein (0.8 mg/kg, once per week). The pain was relieved after 4 weeks of continuous treatment. Two months later, the child did not follow-up and stopped all treatment. On May 12, 2020, the pain in the bilateral hip joints became more severe and was accompanied by occasional headaches. The patient was then admitted to our hospital on May 14 for further examinations. No other symptoms were present (Figure 1).

The girl was diagnosed with immune thrombocytopenia (PLT: 87 × 109/L) in 2017. At that time, cytological examination of the bone marrow showed active bone marrow hyperplasia, maturation disorders of megakaryocytes, and diffused distribution of platelets. The platelet count returned to a normal level after venous infusion of immunoglobulin. No history of organ transplantation or repeated infection was reported. In addition, no remarkable family history was reported.

Upon physical examination, her vital signs were in a normal range with a body weight of 16 kg. The examination of her chest and abdomen showed no obvious abnormalities. No swelling or pressing pain was found in the joints of limbs. However, the 4-shaped sign showed positive results.

During routine laboratory and blood examinations: C-reactive protein (CRP), IL-6, serum ferritin, ASO, and rheumatoid factors (RF) were all within a normal range. Autoimmune antibody (ANCA, ANA, ANA spectrum, PR3-IgG, MPO-IgG, GBM-IgG, and anti-CCP) and pathogenic examination (tuberculosis antibody, tuberculosis infection-specific T cells, mycoplasma antibody, HIV, and fungi) were all negative. EB-VCA-IgG and EB-NA-IgG levels were elevated; EB-VCA-IgM and EB-EA-IgG levels were normal. EBV-DNA quantification showed normal results. Examination of HBsAg, HBeAg, and HBcAb showed positive, and serum quantification of HBV-DNA was 3.38 × 10^7^ IU/ml. The levels of immunoglobulins (A, E, G, and M) and complements C3 and C4 were all within a normal range (Table 1). Lymphocyte subsets were normal.

**Table 1:** Laboratory examination results after hospitalization.

Blood routine test		Anti-nuclear antibodies (ANAs)	Negative
White blood cell (×10^9^/L)	5.45	ANCA	Negative
Neutrophils ratio (%)	0.593	PR3-IgG	Negative
Lymphocyte ratio (%)	0.338	MPO-IgG	Negative
Hemoglobin (g/L)	131	GBM-IgG	Negative
Platelet (×10^9^/L)	109	PPD-IgG/IgM	Negative
Red blood cell (×10^12^/L)	4.55	SPOT-TB test	Negative
AST (IU/L)	26.1	Mp-Ab	Negative
ALT (IU/L)	23.3	HIV-Ab	Negative
Creatinine (μmol/L)	21.6	HBsAg	Positive
Urea nitrogen (mmol/L)	3.52	HBeAg	Positive
CK (U/L)	69.3	HBcAb	Positive
CK-MB (IU/L)	31.7	HBV-DNA (0–100 IU/ml)	3.38 × 10^7^/L
LDH (IU/L)	229.0	EB-VCA-IgG (0–20 U/ml)	>750
C-reactive protein (0–8 mg/L)	2.51	EB-NA-IgG (0–40 U/ml)	<10
IL-6 (<7 pg/mL)	1.74	EB-VCA-IgM (0–40 U/ml)	35.6
Serum ferritin (15–152 ng/mL)	25.4	EB-EA-IgG (0–20U/ml)	48.8
ASO (0–100 IU/ml)	<25	EBV-DNA (0–400 Copies/mL)	<4 × 10^2^
Anti-CCP (0–5 U/ml)	<0.50	Immunoglobulin A (0.14–1.38 g/L)	0.57
Rheumatoid factor (0–20 IU/ml)	<20	Immunoglobulin E (<90 IU/ml)	<5
Galactomannan (<0.5)	0.152	Immunoglobulin G (3.6–10.6 g/L)	7.87
(1,3)-β-D-glucan (0–100.5 pg/ml)	8.2	Immunoglobulin M (0.38–1.44 g/L)	0.40
AFP (0–9 ng/ml)	0.99	C3 complement (0.79–1.52 g/L)	0.74
CEA (0–10 ng/ml)	0.49	C4 complement (0.16–0.38 g/L)	0.26

*AFP, Alpha fetal protein; CEA, Carcinoembryonic antigen; HBV, Hepatitis B virus; HBsAg, hepatitis B virus surface antigen; HBeAg, hepatitis B virus E antigen; HBcAg, hepatitis B virus core antigen; Mp-Ab:Mycoplasma Pneumoniae Antibodies; TB-Ab, tubercle bacillus antibody;EB-VCA-IgG, Epstein-Barr virus capsid antigen IgG; EB-NA-IgG, Epstein-Barr virus nuclear antigen-IgG; EB-EA-IgG, Epstein-Barr virus early antigen-IgG; Ig, Immunoglobulin; ANA, antinuclear antibodies; ANCA, anti-neutrophilic cytoplasmic antibodies; PR3-IgG, proteinase 3-IgG; MPO-IgG, myeloperoxidase-IgG; GBM-IgG, glomerular basal membrane-IgG*.

**Figure 2:**
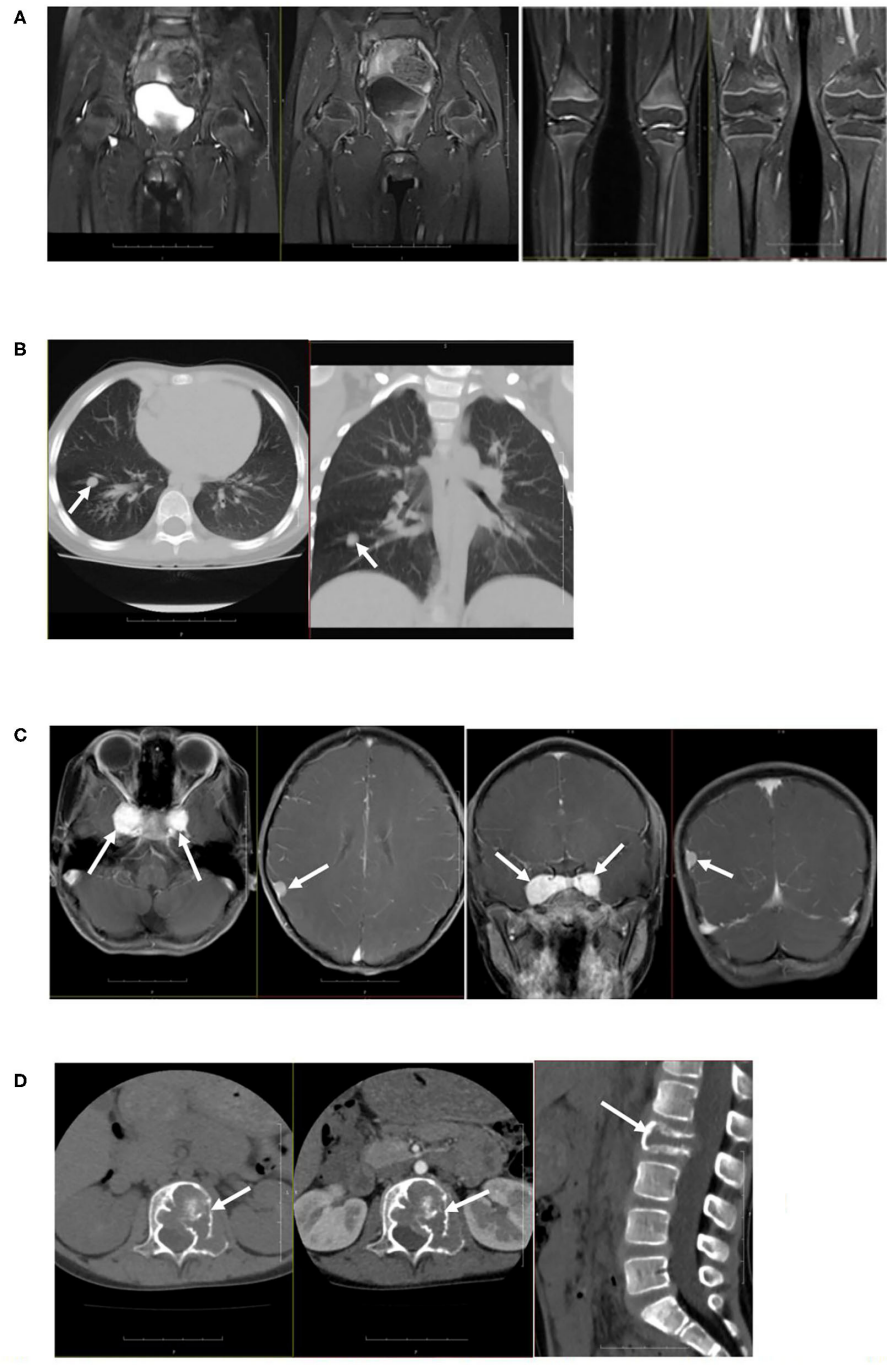
Imaging examination. **(A)** Plain and enhanced MRI scanning of the knee joints and hip joints showed that the synovium of bilateral knee and hip joints were slightly thickened and enhanced, with a small amount of effusion in joints. **(B)** Plain CT scanning of lungs showed multiple nodules and stripe-shaped shadows in bilateral lungs, and the size of the largest shadow was 0.63 × 0.74 cm. **(C)** Plain and enhanced MRI scanning of the skull showed nodular lesions at the parasellar and meninges of the right parietal lobe. **(D)** Plain and enhanced MRI scanning of the spine showed bone mass destruction of the L2 vertebral body and left vertebral pedicle.

Colored ultrasound examination of the retroperitoneum showed no masses or abnormalities. Plain CT scanning of the lungs showed multiple nodules and stripe-shaped shadows bilaterally in the lungs. The largest was 0.63 × 0.74 cm. Plain and enhanced MRI scanning of the knee and hip joints revealed that the synovium of the bilateral knee and hip joints were slightly thickened and enhanced, with a small amount of effusion in the joints. Plain and enhanced CT scanning of the lumbar vertebrae showed the adnexa area left to the L2 vertebral body had osteolytic bone destruction and was accompanied by soft tissue masses. Plain and enhanced MRI scanning of the skull, pituitary, and spine showed nodular lesions at the parasellar and meninges of the right parietal lobe and bone mass destruction of the L2 vertebral body and left vertebral pedicle (Figure 2). Electromyography (EMG) of bilateral lower limbs showed no abnormalities in nerve conduction or muscle contraction.

Bone marrow biopsy examination showed active bone marrow hyperplasia, substantially reduced erythroid hyperplasia, and elevated lymphocytes. Biopsy of the inguinal lymphocytes revealed that the microscopy and immunohistochemistry findings agreed with the changes of reactive hyperplasia of the lymph node.

**Figure 3:**
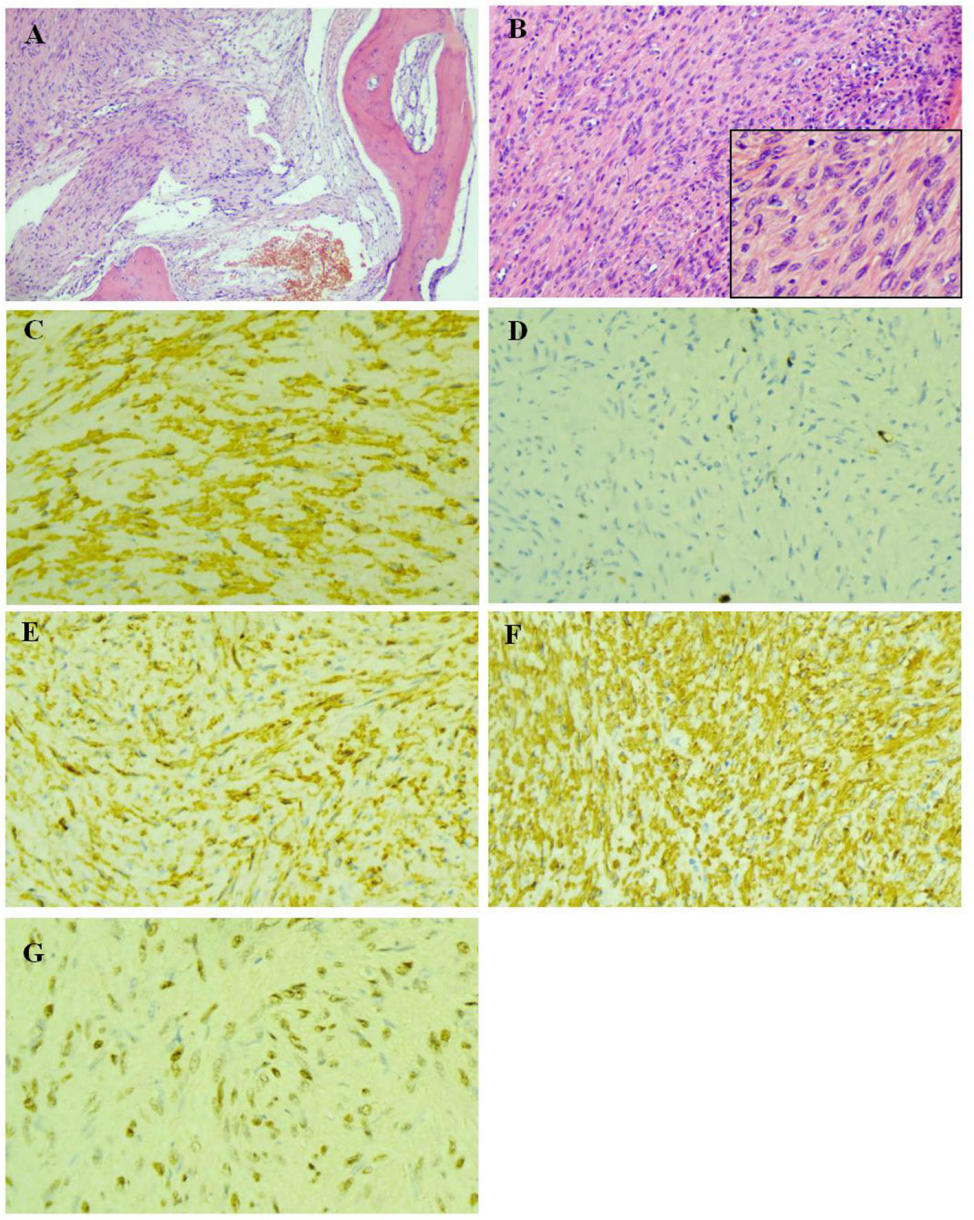
Pathological examination of the biopsy of lesion tissues of the L2 vertebral body. **(A)** The bone tissue were invaded by tumor cells (HE × 100). **(B)** The tumor cells was spindle-shaped, with abundant cytoplasm, red stain and mild atypia, while no evident nuclear fission was observed (HE × 200). **(C)** The actin were expressed in the cytoplasm of tumor cells (IHC × 400). **(D)** The highest ki-67 positive index was 6% (IHC × 400). **(E)** The h-Caldesmon were expressed diffusely in the cytoplasm of tumor cells (IHC × 400). **(F)** SMA were expressed diffusely in the cytoplasm of tumor cells (IHC × 400). **(G)** Epstein-Barr virus encoded RNA *situ* hybridization showed diffusely positive (EBER × 400).

So far, the diagnosis of the child is still not clear. Two biopsies were performed within the L2 vertebral lesion tissues. The first biopsy was not diagnosed because EBER *in-situ* hybridization was not performed. The diagnosis was not made until after the second biopsy of a positive EBER *in-situ* hybridization was performed (Figure 3).

The girl's pathological examination showed EBER (+); thus, she was diagnosed with EBV-SMT. However, she was not previously infected with HIV or had any organ transplantation. In addition, the girl had no history of immunodeficiency-related diseases. In order to clarify the possibility of undiagnosed immunodeficiency or other causes, blood was obtained for whole-exome sequencing. The results showed no related pathogenic genes.

The patient was admitted to the hospital for arthritis. The imaging examinations after admission showed multiple lesions in the skull, lungs, and vertebral body. Other hematological tumors or Langerhans cell histiocytosis were considered during the diagnosis, which was not in agreement with the pathological and other examination results. Biopsy of the L2 vertebral body was then performed to clarify the diagnosis. Finally, the *in-situ* hybridization of the tumor of the lumbar vertebrae suggested non-HIV/transplantation-related EBV-SMT. The patient then received surgery (lumbar lesions resection + fusion bone grafting and internal fixation) without chemo or radiotherapy. The hip pain improved except for occasional headache. The patient is still under regular follow-up now.

## Discussion

SMT was first reported by Pritzker et al. (1) in 1970 in a patient who underwent organ transplantation. However, the causal relationship with EBV was not verified until 1995 (2). To date, studies have demonstrated that patients sensitive to EBV-SMT include those with HIV infection, patients who underwent immunosuppressive therapy after organ transplantation, and those with congenital immunodeficiency (3–5). Although, EBV-SMT can occur in both children and adults, simple SMT in children is exceedingly rare. However, with the prevalence of AID, the occurrence of HIV-SMT in children has increased.

EBV-SMT has become the second leading tumor in AID afflicted children (6). However, the incidence rate of EBV-SMT is still unclear. In a study performed by Stubbins et al. (7) in 2019, 474 children and 4532 adults received organ transplantation at the University of Alberta Hospital between January 1984 and December 2015, and only three developed EBV-SMT. Another study reviewed 975 adult patients who received renal transplantation between 1985 and 2000 and revealed that 16 patients developed EBV-SMT, with an overall incidence of 1.67% (8). Nevertheless, previous studies were all single-center studies of single disease types. So far, no large-scale study has been performed to investigate the overall incidence of the disease.

With an increasing understanding of EBV-SMT, more and more cases of EBV-SMT have been reported. However, most cases were immunocompromised patients, while EBV-SMT patients with autoimmune diseases have been rarely reported. In 2019, So et al. (9) reported a case of a 49-year-old female patient with idiopathic inflammatory myopathy, which was also the first reported case of an autoimmune disease accompanied by EBV-SMT. In this study, we reported the first case of a patient with JIA associated with EBV-SMT. Unlike previously reported cases, our patient was incredibly young and received short-term immunosuppressive therapy; the EBV-SMT occurred secondary to JIA. However, it remains unclear whether EBV-SMT was associated with the application of an immunosuppressant agent, the state of JIA, or a dormant EBV activation after infection. Interestingly, Au et al. (10) found that treating autoimmune diseases with azathioprine or cyclophosphamide could induce EBV-negative lymphoproliferative disorders, suggesting that drugs could directly induce lymphoproliferative disorders. On the other hand, Wang et al. (11) reported a 31-year-old non-HIV/organ transplantation/immunodeficiency patient who was pathologically confirmed with multiple intestinal EBV-SMT. Their data demonstrated that EBV-SMT could also occur in patients with normal immune functions (11). The above findings demonstrated that the pathogeneses of EBV-SMT involve multiple pathways and mechanisms.

The exact pathogenesis of EBV-SMT is still unclear. Currently, it is well-known that EBV infection is closely associated with immune suppression. For instance, negative serum EBV before transplantation, idiopathic EBV infection after transplantation, and high viral load have been identified as independent risk factors of post-transplantation EBV-SMT (7). In addition, more potent immunosuppression, such as thymectomy, could also be a risk factor inducing EBV-SMT (7). However, the exact mechanisms involved in the invasion of EBV into smooth muscle cells and the consequent proliferation and transformation remain poorly understood. With the advancement of EBV-SMT research over recent years, several studies reported that the pathogeneses of EBV-SMT could be associated with the infection of smooth muscle cells and a tumor transformation induced by cloning and amplification of EBV (12). Nonetheless, the invasion of EBV into smooth muscle cells is still not fully understood. Previous studies showed a positive expression of EBV receptor CD21 receptor in patients with HIV-SMT, suggesting that EBV could invade smooth muscle cells *via* this pathway (13). However, the CD21 receptor staining of the SMT patient after transplantation was negative, suggesting that there are other pathways involved in SMT occurrence. For instance, the infection could occur by fusing smooth muscle cells with the EBV-infected lymphocytes, which needs to be further investigated (2, 14). Still, the transformation and proliferation of EBV after invading smooth muscle are of great importance and should be addressed by future studies.

In conclusion, EBV-SMT is a rare tumor lacking specificity in clinical practices; thus, it could be easily misdiagnosed as other tumorous diseases. The diagnosis of EBV-SMT relies on pathological examinations and EBER *in-situ* hybridization, while tumor resection and discontinuation of immunosuppression are still the main treatment approaches for this disease. Herein, we reported a rare case that suggested how EBV-SMT can occur secondary to immune diseases not only in patients with AID, after organ transplantation, or congenital immunodeficiency. Unfortunately, whether the occurrence of EBV-SMT in this case was related to the application of immunosuppressant agent, the state of JIA, or a dormant EBV activation after infection is unknown. The interaction among them needs further study.

## Data Availability Statement

The original contributions presented in the study are included in the article/supplementary material, further inquiries can be directed to the corresponding author/s.

## Ethics Statement

The studies involving human participants were reviewed and approved by the Institutional Review Board of the Hunan Children's Hospital. Written informed consent to participate in this study was provided by the participants' legal guardian/next of kin. Written informed consent was obtained from the minor(s)' legal guardian for the publication of any potentially identifiable images or data included in this article.

## Author Contributions

ZK, JX, and ZL contributed to conception and design of the study. JX organized the data. ZK wrote the first draft of the manuscript. ZL reviewed sections of the manuscript. All authors contributed to manuscript revision, read, and approved the submitted version.

## Conflict of Interest

The authors declare that the research was conducted in the absence of any commercial or financial relationships that could be construed as a potential conflict of interest.
